# Etablierung gesundheitsfördernder Strukturen in der stationären Langzeitversorgung durch Digitalisierung: Aufruf zu einem Perspektivwechsel

**DOI:** 10.1007/s00103-023-03686-4

**Published:** 2023-04-15

**Authors:** Florian Fischer, Cordula Endter

**Affiliations:** 1 Bayerisches Zentrum Pflege Digital, Hochschule Kempten, Albert-Einstein-Straße 6, 87437 Kempten, Deutschland; 2 Katholische Hochschule für Sozialwesen Berlin, Berlin, Deutschland

**Keywords:** Prävention, Gesundheitsförderung, Langzeitpflege, Organisationsentwicklung, Innovation, Prevention, Health promotion, Long-term care, Organization development, Innovation

## Abstract

Die Digitalisierung nimmt im Bereich der Gesundheitsförderung und Prävention einen immer höheren Stellenwert ein. Gleichzeitig hat das Präventionsgesetz zu einer Stärkung von Interventionen der Gesundheitsförderung und Prävention in Settings beigetragen. In einem lebenswelt- bzw. settingorientierten Ansatz werden Maßnahmen der Prävention und Gesundheitsförderung sowohl auf das Verhalten als auch die Verhältnisse ausgerichtet. Obwohl im Setting der „Arbeitswelt“ in den vergangenen Jahren der Einsatz eines digitalen betrieblichen Gesundheitsmanagements zugenommen hat, werden die Potenziale der Digitalisierung nur unzureichend genutzt. Dies gilt insbesondere für die Gesundheitsförderung und Prävention bei Pflegefachkräften.

Daher wird in diesem Diskussionsbeitrag der Fokus auf Gesundheitsförderung und Prävention in Einrichtungen der stationären Langzeitpflege gelegt. Da die strukturierte Implementierung digitaler Applikationen in Pflegeeinrichtungen eine Innovation innerhalb der Organisation darstellt, könnte dieses Momentum dazu genutzt werden, zugleich Strukturen und Prozesse gesundheitsförderlich zu gestalten. Dabei wird die Gesundheitsförderung durch Digitalisierung als organisationale Aufgabe mit einer bedarfssensiblen und diversitätsorientierten Ausrichtung verstanden. Somit kann die Digitalisierung ein Vehikel darstellen, um die Etablierung gesundheitsfördernder Einrichtungen in der Langzeitpflege substanziell voranzubringen.

## Abstract (en)

Digitization is becoming increasingly important in health promotion and disease prevention. At the same time, the Prevention Act has contributed to the strengthening of health promotion and disease prevention interventions in various settings. In a lifeworld- or setting-oriented approach, disease prevention and health promotion target both the behavior and the situation around individuals. Although the use of digital occupational health management has increased in working environments in recent years, the potential of digitization is still insufficiently exploited. This is particularly true for health promotion and disease prevention among nursing professionals.

Therefore, this discussion paper focuses on health promotion and disease prevention in inpatient long-term care facilities. Since the structured implementation of digital applications in care facilities represents an innovation within the organization, this momentum could be used to simultaneously design structures and processes in a way that promotes health. In this context, health promotion through digitization is understood as an organizational task with a needs-sensitive and diversity-oriented focus. Digitization can thus be a vehicle for substantially advancing the establishment of health-promoting facilities in long-term care.

## Einleitung

Geht es um die Potenziale von digitaler Unterstützung im Kontext der Pflege, so liegt der Fokus entweder auf Aspekten der betrieblichen Verwaltung, Dokumentation und Organisation von Arbeitsprozessen (sowohl als Praktiken zwischen Menschen als auch technische Unterstützung nicht-menschenbezogener Unterstützungshandlungen) in Pflegeeinrichtungen [1, 2] oder aber auf dem direkten Pflegegeschehen (z. B. Telepflege oder Pflegerobotik) und damit auf der Interaktion von Pflegefachkräften bzw. pflegenden Angehörigen einerseits und den zu Pflegenden andererseits. Die treibende Annahme ist dabei, dass Pflegende – seien es professionelle Pflegefachkräfte oder pflegende Angehörige – mittels Technologie in ihren Pflegehandlungen unterstützt, entlastet, angeleitet oder gemonitort werden, so dass die pflegerische Versorgung sichergestellt ist. Trotz der zahlreichen Innovationen und sowohl politischen wie auch organisationalen Bemühungen, die Nutzung digitaler Technologien in der Pflege voranzubringen, ist dieser Bereich weiterhin ausbaufähig [3]. In diesem Zusammenhang wurde bereits vielfach die sozialethische Frage diskutiert, inwieweit Institutionen der Altenhilfe und -pflege im Spannungsfeld zwischen betrieblicher Organisation und sozialem Auftrag Digitalisierung nutzen dürfen, können oder gar sollen, um Klient:innen in ihrer Lebenswelt zu unterstützen [4]. Auch Fragen der Technikakzeptanz und -kompetenz sowohl aufseiten der Pflegenden [5] als auch älterer Menschen bzw. der zu Pflegenden [6] standen im Blickpunkt der Betrachtung.

Im Rahmen dieses Diskussionspapiers wollen wir nun einen Perspektivwechsel wagen, welcher zumindest schon teilweise Anklang in der aktuellen Diskussion in Forschung und Praxis gefunden hat: Dabei geht es um die Frage, inwieweit digitale Angebote zur Prävention und Gesundheitsförderung für professionelle Pflegefachkräfte in stationären Settings ein Mittel sein können, um die Akzeptanz sowie die positive Assoziation mit Technik und Digitalität zu fördern, indem es gerade nicht um dyadische Pflegesituationen, sondern um die eigene Gesundheit geht. Dahinter steht die Annahme, dass Digitalisierung mehr als eine Intervention auf Verhaltensebene ist (Bsp.: App zur Bewegungsförderung), sondern es gezielt einer Verhältnisorientierung (sprich einer Veränderung der den Menschen umgebenden Faktoren) bedarf. In einem lebenswelt- bzw. settingorientierten Ansatz werden Maßnahmen der Prävention und Gesundheitsförderung sowohl auf das Verhalten als auch die Verhältnisse ausgerichtet [7]. In diesem Zusammenhang können digitale Applikationen zur Entwicklung gesundheitsförderlicher Lebens- und Arbeitsbedingungen beitragen [8] und darin – quasi sekundär – auch die Akzeptanz gegenüber anderen digitalen Angeboten und Anwendungen verbessern und die Aneignung digitaler Kompetenzen bzw. die Motivation zum Kompetenzaufbau befördern [9, 10].

Zudem können Pflegefachkräfte in ihrer Rolle als Multiplikator:innen einen starken Einfluss auf die kompetente Nutzung digitaler Technologien bei Pflegebedürftigen haben. Zentral hierfür sind eine positive Einstellung gegenüber digitalen Technologien und deren aktive Nutzung. Entsprechend gilt es zu untersuchen, inwieweit Gesundheitsförderung hier nicht nur ein Auftrag mit Blick auf die zu Pflegenden, sondern auch auf das Personal selbst ist.

Hierzu bedarf es einer systemisch-organisationalen Perspektive, in welcher die Expertise aus verschiedenen Disziplinen – insbesondere Public Health und Pflegewissenschaft – zusammengebracht wird. Im Folgenden wird dies am Beispiel der Digitalisierung und Gesundheitsförderung und Prävention in der stationären Langzeitversorgung aufgezeigt. Dabei wird die Notwendigkeit zur Etablierung gesundheitsfördernder Einrichtungen in der Langzeitpflege hervorgehoben, in welchen Gesundheitsförderung durch Digitalisierung als organisationale Aufgabe mit einer bedarfssensiblen und diversitätsorientierten Ausrichtung verstanden wird.

## Digitalisierung in Gesundheitsförderung und Prävention

Die Digitalisierung nimmt im Bereich der Gesundheitsförderung und Prävention einen immer höheren Stellenwert ein [11]. Sie verändert und erweitert das Spektrum der Instrumente im Bereich der Gesundheitsförderung und Prävention. Erweiterte Möglichkeiten ergeben sich insbesondere im Kontext der (Gesundheits‑)Kommunikation, der datengestützten Adaptation von Maßnahmen, der Daten- und Bedarfserhebung zu Präventionspotenzialen und der Evaluation von Interventionen. Dabei ist die Digitalisierung kein kurzfristiger Trend, sondern eine mittel- und langfristige Entwicklung, welche größere Flexibilität und gezieltere Anpassung an Bedarfe und Bedürfnisse der Nutzer:innen(gruppen) entsprechender digitaler Applikationen auf der einen Seite ermöglicht, aber auf der anderen Seite auch erfordert.

In Deutschland hat das Inkrafttreten des Präventionsgesetzes im Jahr 2015 zu einer Stärkung von Maßnahmen und Interventionen der Gesundheitsförderung und Prävention in Settings geführt, mit denen förderliche Lebenswelten entstehen sollen, die zur Gesunderhaltung der in den Settings lebenden oder arbeitenden Menschen beitragen. Gegenwärtig werden digitale Anwendungen jedoch zumeist in der Verhaltensprävention genutzt, um individuelle gesundheitsrelevante Verhaltensweisen zu adressieren. Eine Verhältnisprävention unter Einsatz der Digitalisierung wäre jedoch möglich, sofern entsprechende digitale Anwendungen an Strukturen und Rahmenbedingungen im Setting ansetzen (z. B. durch digitale Plattformen oder Apps zur Vernetzung) und somit zu einer Veränderung der Lebens- und Arbeitsumwelt führen [12]. Ein solcher Einsatz der Digitalisierung würde somit sowohl auf den Handlungsfeldern zur Gesundheitsförderung der Ottawa-Charta [7] basieren als auch die im Präventionsgesetz geforderten Paradigmen aufgreifen, um sowohl äußere als auch verhaltensabhängige Risiken und Belastungen im Hinblick auf (spezifische) Krankheitsvermeidung zu senken, indem persönliche und kollektive Ressourcen (z. B. über kontext- oder settingbezogene Maßnahmen) gestärkt werden.

### Digitalisierung im betrieblichen Gesundheitsmanagement

Insbesondere bei digitalen Interventionen sind verhaltens- und verhältnisbezogene Maßnahmen nicht immer klar voneinander zu trennen: Erfolgreiche Maßnahmen sollten diese beiden Interventionsformen eng miteinander verzahnen [13]. Im Setting „Arbeitswelt“ hat diesbezüglich in den vergangenen Jahren der Einsatz eines digitalen betrieblichen Gesundheitsmanagements zugenommen [14]. Betriebliches Gesundheitsmanagement wird als „die Entwicklung betrieblicher Strukturen und Prozesse verstanden, welche die gesundheitsförderliche Gestaltung von Arbeit und Organisation und die Befähigung zum gesundheitsfördernden Verhalten der Mitarbeiterinnen und Mitarbeiter zum Ziel haben“ [15]. Grundgedanke dabei ist, dass die Verankerung von Gesundheit als betriebliches Ziel und Querschnittsaufgabe in allen Leitungsfunktionen mit Hilfe von Managementstrategien erreicht werden kann und soll. Dazu gehört die systematische Gestaltung von gesundheitsförderlichen innerbetrieblichen Strukturen und Prozessen [16], da gesunde Organisationen (z. B. Unternehmen) das Wohlbefinden und die Produktivität ihrer Mitarbeiter:innen fördern und somit auch den Unternehmenszielen dienen [17]. Digitale Tools werden bislang jedoch nur von wenigen Unternehmen im betrieblichen Gesundheitsmanagement verwendet [14] – dies gilt auch und insbesondere für den Gesundheits- und Pflegebereich. Zur Förderung der Umsetzung von betrieblichem Gesundheitsmanagement haben z. B. das internationale Netzwerk gesundheitsförderlicher Krankenhäuser der Weltgesundheitsorganisation (WHO) und die nationalen Netzwerke gesundheitsfördernder Krankenhäuser in Deutschland und Österreich wirkungsvolle Strategien ausgearbeitet [18]. Dies erfolgte jedoch noch ohne Berücksichtigung der Möglichkeiten, welche die Digitalisierung in diesem Kontext bietet.

### Umsetzung in der stationären Langzeitversorgung

Obwohl Prävention und Gesundheitsförderung seit jeher Teil der Ausbildung und des Selbstverständnisses in der langzeitstationären Pflege älterer Menschen sind, werden deren Potenziale in der Versorgungspraxis (auch unabhängig von der Digitalisierung) nicht oder nur sehr begrenzt genutzt [19]. Obwohl sich in der Langzeitpflege – wie in vielen anderen Lebens- und Arbeitswelten auch – zwar immer mehr die Grundgedanken einer Gesundheitsförderung im Setting durchsetzen, bedarf es immer noch vielmehr einer gesundheitsfördernden (Organisations‑)Kultur, die sämtliche Stakeholder (in diesem Fall Bewohner:innen, Angehörige und Mitarbeiter:innen) berücksichtigt. Insbesondere in der stationären Langzeitpflege zeigt sich zugleich die Diskrepanz eines hohen Bedarfs an entsprechenden Strategien und einer geringen Umsetzung digitaler Anwendungen als Instrument des betrieblichen Gesundheitsmanagements. Da sich Gesundheit am besten im Setting des Arbeitsplatzes über erfolgreich durchgeführte Projekte – und somit durch Integration entsprechender Angebote bzw. gesundheitsförderlicher Strukturen und Prozesse in die Organisationsentwicklung – fördern lässt [20], stellt sich die Frage, inwieweit Digitalisierungsansätze einen Anreiz für eine systematische Organisations- und Personalentwicklung und die Ausprägung gesundheitsfördernder Strukturen in Pflegeeinrichtungen darstellen können.

## Etablierung gesundheitsfördernder Einrichtungen in der Langzeitpflege durch digitale Transformation

Die digitale Transformation hat auch die (pflegerische) Versorgung älterer Menschen längst erreicht [21]. Spätestens die Coronapandemie hat zu einer solchen Disruption geführt, dass nicht nur Reaktionen auf sich verändernde Ausgangsbedingungen notwendig sind (u. a. im Hinblick auf demographischen Wandel und Fachkräftemangel in der Pflege), sondern es eines aktiven Gestaltens bedarf, in welchem die Potenziale der Digitalisierung genutzt und zugleich die damit verbundenen Herausforderungen bewältigt werden. Zur erfolgreichen Umsetzung des Digitalisierungsprozesses in Pflegeeinrichtungen ist eine klare Strategie erforderlich, die aus den Einrichtungen selbst kommen muss. Wie in anderen Branchen mangelt es hieran jedoch häufig noch, da zwar der potenzielle Nutzen der Digitalisierung erkannt wird, aber Schwierigkeiten sowohl bei der unmittelbaren Einführung als auch langfristigen Nutzung bestehen. Die erforderlichen Veränderungsprozesse müssen unmittelbar an bestehende Ablauforganisationen ansetzen, sodass vielfach eine Neuausrichtung der Art und Weise, wie Pflegereinrichtungen in diesem Bereich in Zukunft agieren wollen, notwendig erscheint [3].

### Gesundheitsförderung durch Digitalisierung als organisationale Aufgabe

Aus unserer Perspektive wird die Entwicklung gesundheitsfördernder Strukturen in Pflegeeinrichtungen in Gegenwart und Zukunft nicht mehr ohne Berücksichtigung der Auswirkungen der Digitalisierung funktionieren können. Die Gestaltung der digitalen Transformation sollte somit Teil der Organisationsentwicklung sein. Mit Blick auf die Gesundheit der Beschäftigten – und auch der Pflegebedürftigen – ist in Pflegeeinrichtungen eine Veränderung der „Versorgungskultur“ erforderlich, die sich von der reinen „Verwahrung“ der Bewohner:innen mit einer Konzentration auf die Defizitorientierung hin zu einer Ressourcenorientierung unter Nutzung von (ggf. digital unterstützten) Maßnahmen der Prävention und Gesundheitsförderung wandelt [22]. Ziel sollte daher die Entwicklung einer gesundheitsfördernden Organisationskultur [15] sein, zu der auch eine systematische Personalentwicklung (z. B. durch Qualifikations- und Kompetenzentwicklung der Beschäftigten) gehört.

### Bedarfssensible und diversitätsorientierte Ausrichtung

Die Integration der Digitalisierung in den Arbeitsalltag in Pflegeeinrichtungen ist aber voraussetzungsreich, v. a. aufgrund der Heterogenität der beteiligten Akteure, der individuellen und komplexen Versorgungssituationen sowie der Vielschichtigkeit der direkten und indirekten – sowie intendierten und nicht-intendierten – Auswirkungen des Einsatzes technischer Innovationen. Ein erfolgreicher Umgang mit der digitalen Transformation der Pflege erfordert somit mehr als die reine Implementierung von Technologien. Digitale Anwendungen sind im Kontext der Pflege vielmehr komplexe Interventionen [12], welche einer kontinuierlichen Begleitung, Reflexion und Evaluation bedürfen.

Die explizite Berücksichtigung von Gesundheitsförderung und Prävention im Setting der Pflegeeinrichtungen erfolgt mit doppeltem Mandat: sowohl für die Beschäftigten als auch (un)mittelbar für die Pflegebedürftigen. Umso wichtiger erscheint es, nicht nur Gesundheitsförderung in der Langzeitpflege zu fordern und zu fördern – im Sinne von projektförmigen und somit punktuellen Implementierungen konkreter Einzelmaßnahmen –, sondern das durchaus anspruchsvollere Ziel anzustreben, Pflegeeinrichtungen insgesamt und umfassend zu einer gesundheitsfördernden Organisation zu entwickeln. Erforderlich sind dafür der Aufbau bzw. die nachhaltige Stärkung von Strukturen (z. B. Gesundheitsförderungsbeauftragte) und Prozessen (z. B. Implementierungsprojekte) mittels entsprechender personeller, zeitlicher und finanzieller Kapazitäten.

Die strukturierte Implementierung digitaler Applikationen in Pflegeeinrichtungen ist eine Innovation innerhalb der Organisation. Dieses Momentum kann gleichzeitig genutzt werden, um Strukturen und Prozesse gesundheitsförderlich zu gestalten. Arbeitsbelastungen können reduziert werden – sei es zum Beispiel durch eine sprachgesteuerte oder teilautomatisierte Pflegedokumentation oder auch digitale Applikationen zum Stressmanagement –, wodurch die Mitarbeiter:innenzufriedenheit gesteigert wird [23]. Dies kann wiederum beim Pflegepersonal positive Auswirkungen auf die Akzeptanz digitaler Anwendungen und die Bereitschaft zu deren Nutzung haben. Daraus schlussfolgernd ergeben sich Synergien aus der gelingenden Implementierung einer digitalen Anwendung in der Pflege, sofern dieser Prozess als Teil der Organisationsentwicklung und der Schaffung gesundheitsfördernder Strukturen und Prozesse in Pflegeeinrichtungen betrachtet wird. Somit kann die Digitalisierung ein Vehikel darstellen, um die Etablierung gesundheitsfördernder Einrichtungen in der Langzeitpflege substanziell voranzubringen und damit weiterführend auch Akzeptanz und Nutzung digitaler Technologien und Anwendungen in der Pflege zu fördern.

## Fazit

Sowohl die Förderung der Digitalisierung als auch die Schaffung gesundheitsförderlicher Strukturen in Pflegeeinrichtungen stellen jeweils Innovationen dar. Aus beiden Maßnahmen können Synergieeffekte entstehen, sofern diese Interventionen nicht auf der Ebene von Einzelmaßnahmen verbleiben, sondern Teil strukturierter, systemisch-organisationaler Veränderungsprozesse in den Pflegeeinrichtungen sind und nachhaltig verankert werden. Damit verschiebt sich der Fokus von primär personenzentrierten auf organisationale Ansätze in der Betrachtung von Gesundheit und Digitalisierung. Diese Neuausrichtung wird durch die gesetzlich geförderten Maßnahmen der Digitalisierung in Gesundheit und Pflege, wie zum Beispiel den Ausbau der Telematikinfrastruktur, verstärkt. Dass Akzeptanz und Kompetenzaufbau aufseiten des Personals hier allein nicht reichen, sondern es ebenso entsprechender IT-Infrastrukturen und einer Ausgestaltung von Prozessen der pflegerischen Mensch-Technik-Interaktion bedarf, wird dabei ebenfalls deutlich. Daraus ergibt sich die Notwendigkeit einer Weitung der Forschungsperspektive. Ging es bislang vorrangig um Nutzer:innen, wie beispielsweise Pflegefachkräfte, pflegende Angehörige oder Pflegebedürftige, sollten zukünftig stärker organisationale und lebensweltorientierte Aspekte Berücksichtigung finden. Die sich daraus ergebenden Anforderungen für Forschung und Praxis können nur von Vertreter:innen aus Public Health und Pflege gemeinsam bewältigt werden.
